# Modification of Polyhydroxyalkanoates Polymer Films Surface of Various Compositions by Laser Processing

**DOI:** 10.3390/polym15030531

**Published:** 2023-01-19

**Authors:** Ekaterina I. Shishatskaya, Natalia O. Zhila, Alexey E. Dudaev, Ivan V. Nemtsev, Anna V. Lukyanenko, Tatiana G. Volova

**Affiliations:** 1 Department of Medical Biology, School of Fundamental Biology and Biotechnology, Siberian Federal University, Svobodnyi Av., 79, 660041 Krasnoyarsk, Russia; 2 Institute of Biophysics SB RAS, Federal Research Center “Krasnoyarsk Science Center SB RAS”, 50/50 Akademgorodok, 660036 Krasnoyarsk, Russia; 3 Chemistry Engineering Centre, ITMO University, Kronverkskiy Prospekt, 49A, 197101 Saint Petersburg, Russia; 4 Basic Department of Biotechnology, School of Fundamental Biology and Biotechnology, Siberian Federal University, Svobodnyi Av., 79, 660041 Krasnoyarsk, Russia; 5 L.V. Kirensky Institute of Physics SB RAS, Federal Research Center “Krasnoyarsk Science Center SB RAS”, 660036 Krasnoyarsk, Russia; 6 Federal Research Center “Krasnoyarsk Science Center of the Siberian Branch of the Russian Academy of Sciences”, 660036 Krasnoyarsk, Russia; 7 Institute of Engineering Physics and Radio Electronics, Siberian Federal University, 660041 Krasnoyarsk, Russia

**Keywords:** PHAs, polymer films, CO_2_ laser, laser processing, SEM, AFM, water contact angles, fibroblast NIH 3T3, MTT test

## Abstract

The results of surface modification of solvent casting films made from polyhydroxyalkanoates (PHAs) of various compositions are presented: homopolymer poly-3-hydroxybutyrate P(3HB) and copolymers comprising various combinations of 3-hydroxybutyrate (3HB), 3-hydroxyvalerate (3HV), 4-hydroxybutyrate(4HB), and 3-hydroxyhexanoate (3HHx) monomers treated with a CO_2_ laser in continuous and quasi-pulsed radiation modes. The effects of PHAs film surface modification, depending on the composition and ratio of monomers according to the results of the study of SEM and AFM, contact angles of wetting with water, adhesion and growth of fibroblasts have been revealed for the laser radiation regime used. Under continuous irradiation with vector lines, melted regions in the form of grooves are formed on the surface of the films, in which most of the samples have increased values of the contact angle and a decrease in roughness. The quasi-pulse mode by the raster method causes the formation of holes without pronounced melted zones, the total area of which is lower by 20% compared to the area of melted grooves. The number of viable fibroblasts NIH 3T3 on the films after the quasi-pulse mode is 1.5–2.0 times higher compared to the continuous mode, and depends to a greater extent on the laser treatment mode than on the PHAs’ composition. The use of various modes of laser modification on the surface of PHAs with different compositions makes it possible to influence the morphology and properties of polymer films in a targeted manner. The results that have been obtained contribute to solving the critical issue of functional biodegradable polymeric materials.

## 1. Introduction

The intensive use of non-destructible synthetic plastics and their accumulation in the biosphere is a global environmental problem [1,2], the solution of which is a gradual transition to degradable materials [3,4]. Among degradable polymeric materials, a special place belongs to polyhydroxyalkanoates (PHAs)—polymers of microbiological origin. Polyhydroxyalkanoates are a class of biocompatible and biodegradable thermoplastics with different physicochemical structures and properties [5,6,7,8,9,10,11,12]. They can be processed into various products by almost all available technical methods (casting, emulsification, extrusion, pressing, electrostatic molding, etc.) [13,14], and it is also possible to obtain composite materials based on PHAs with various fillers [15]. The areas of application of PHAs are diverse, from agriculture and urban economy to high-tech biomedicine and pharmaceutical production [16,17,18,19], and they also play a significant role in the “circular economy” [20]. The excellent biocompatibility of polyhydroxyalkanoates at all levels of organization of living matter (cellular, tissue, and organism), long-term and directed resorption in vivo puts forward this class of biopolymers as the most promising for biomedicine. PHAs are especially promising in personalized medicine and cell and tissue engineering [21,22,23,24,25,26]. This is due to the possibility of targeted biosynthesis of PHAs with different chemical compositions and physical properties (strength, hydrophobic–hydrophilic balance, and surface architecture of the formed products).

The first discovered and massively studied representative of PHAs is (poly-3-hydroxybutyrate, P(3HB))—a homopolymer of 3-hydroxybutyric acid. Despite the biodegradability, the application potential of this representative is sharply limited due to the high crystallinity (above 70%) and hydrophobicity, the tendency to “physical aging”, and disordered crystallization [27,28]. In this regard, the properties of polymers, including P(3HB), can be influenced and improved by biological, chemical, or physical methods, such as the biosynthesis of PHAs’ copolymers of various compositions, the use of various fillers for the manufacture of composites based on P(3HB), or processing various chemical reagents, as well as modifications using physical methods, such as liquid plasma treatment, protonation, laser action, etc. [15,24,29,30,31,32,33,34,35]. The use of these methods makes it possible to purposefully modify the properties of polymers, such as changing the hydrophilicity and surface structure, increasing the rate of biodegradation, increasing flexibility and strength, changing the gas-dynamic properties of products, or improving their permeability.

Methods of laser action for the modification of polymer products have been used relatively recently. The main advantage of this method lies in the selective nature of the change in the surface properties of the product without violating the integrity of the material itself and without the accumulation of toxic substances. The papers on this topic describe the results of using various types of lasers (gas, solid-state, semiconductor, etc.) to modify the surface of various biopolymers—polylactide, polycaprolactone, polyglycolide, chitosan, etc., by various methods (ablation, engraving, welding, microrelief, etc.) [34,35,36,37,38].

The effect of laser processing is determined by the type and composition of the material, laser parameters, and the processing mode [39]. Depending on the irradiation mode, as a result of laser ablation (the process of removing material under the action of a laser beam), specific zones are formed on the treated surface that differ significantly from the original ones, and these changes may consist of a change in the relief, such as the appearance of cavities and perforations on the surface. Laser ablation is widely used for various material processing (metals, ceramics, glass and polymers). Modification of the surface of polymers with a laser for medical applications has great prospects, since the surface becomes more developed, which has a good effect on the activity of cells in contact with it [33,35,40,41,42].

There are few studies characterizing the potential of laser surface treatment of PHAs polymer products. Using homogeneous poly-3-hydroxybutyrate P(3HB) as an example, the possibility of changing the surface and bulk properties of products made from this polymer by laser treatment has been shown [43,44,45,46,47]. The authors of [35] describe the results of processing the Picosecond Laser Ablation of films obtained from P(3HB) and medium-chain copolymer poly(3-hydroxyoctanoate)-co-(3-hydroxydecanoate) and show changes in the surface topography. The works [34,48,49] describe the use of the Nd:YAG laser and KrF excimer laser for processing solvent casting films obtained from a copolymer of 3-hydroxybutyrate with 3-hydroxyvalerate, which provides a change in topography, including pore formation and a change in roughness.

Due to the fact that films and membranes made from PHAs are transparent in the visible and near IR spectral regions, lasers generating radiation with a wavelength in the far IR (for example, CO_2_ laser) or ultraviolet spectral regions are suitable for their processing (an excimer laser, such as a Ar–F, Kr–F_2_–Ne gas mixture laser) [44]. In [50], the results of using a CO_2_ laser in continuous and quasi-pulse modes for processing films obtains four types of PHAs—poly-3-hydroxybutyrate and three copolymers of 3-hydroxybutyrate with 4-hydroxybutyrate, 3-hydroxyvalerate, and 3-hydroxyhexanoate (the content of the second monomer in all samples is close to 30 mol.%) are described. Treatment in both regimes leads to various modifications of films depending on their composition and irradiation mode. For each regime, differences in the modification of the surface of the films depending on their composition are revealed. Under constant irradiation with vector lines, melted regions in the form of grooves are formed on the surface of the films. The quasi-pulse mode by the raster method is accompanied by the formation of dimples without pronounced melted zones. The processing mode influences the characteristics of the surface of the films, as well as the number of viable fibroblasts cultivated on the films. Despite the fact that the content of the second monomers in the copolymer samples is close, the resulting effects of irradiation differ (i.e., laser treatment has a different effect on changes in the surface of PHAs films of various compositions).

PHAs is a family of polymers of different chemical compositions, in which, depending on the conditions of biosynthesis, the set and ratio of monomers in the C-chain can vary over a wide range, which affects the properties of the polymers themselves, as well as the properties of the resulting polymer products [7,8,9,10,11,12]. This is clearly illustrated by the results of [51], in which the surface structure and properties of cast films obtained from various PHAs copolymers contain monomers of 3-hydroxybutyrate, 3-hydroxyvalerate, 4-hydroxybutyrate, and 3-hydroxyhexanoate in various proportions. Depending on the content of monomers other than 3-hydroxybutyrate in PHAs (from 10 to 50 mol.% and more) the porosity, contact angle, surface roughness, and mechanical properties of the films vary significantly.

In the present work, for the first time, the effect of laser treatment is researched towards the microstructure, surface properties, and biological properties of films obtained from various types of PHAs copolymers: P(3HB-co-3HV), P(3HB-co-4HB), and P(3HB-co-3HHx), in which the ratio of monomers differ significantly. As noted above, despite the fact that research on laser processing for the modification of various materials is very active, in relation to PHAs, these studies are very limited. In this case, P(3HB) homopolymer and P(3HB-co-3HV) copolymers with a low monomer content, about 8–11 mol.%, have been studied [34,48,49]. In our recent work, the results of laser treatment of four types of PHAs (P(3HB) homopolymer and three copolymers P(3HB-co-3HV), P(3HB-co-4HB), and P(3HB-co-3HHx) with a higher, at about 30 mol.%, but similar content of monomers 3HV, 4 HB, and 3HHx [50]. Due to the fact that the ratio of monomers in PHAs copolymers significantly affects the properties, in particular the degree of crystallinity and the crystallization kinetics of melts, the present article aims to investigate how the content of second monomers in copolymers 3HV, 4HB, 3HHx, and other than 3HB affects the consequences of laser processing.

## 2. Materials and Methods

### 2.1. Material

A number of polyhydroxyalkanoates with different quantitative and qualitative compositions of incoming monomers have been studied in this work (Table 1): 3-hydroxybutyrate homopolymer P(3HB) [-O-CH(CH_3_)-CH_2_-CO-] and copolymers containing the 3HB monomer and another monomer in various proportions. The second monomers differ in chemical structure and C-chain length: 4-hydroxybutyrate (4HB) [-O-CH_2_-CH_2_-CH_2_-CO-], 3-hydroxyvalerate (3HV) [-CH(C_2_H_5_)-CH_2_-CO-], and 3-hydroxyhexanoate (3HHx) [-O-CH(C_3_H_7_)-CH_2_-CO-]. The biosynthesis of polyhydroxyalkanoates has been carried out using the bacterial strain Cupriavidus necator B-10646 according to our own developed technology [52]. 

The culture Cupriavidus necator B-10646 has been grown in the mineral Schlegel medium [53]—a strong phosphate-buffered solution with the following composition: Na_2_HPO_4_⋅H2O − 9.1; KH_2_PO_4_ − 1.5; MgSO_4_⋅H_2_O − 0.2; Fe_3_C_6_H_5_O_7_⋅7H_2_O − 0.025; CO(NH_2_)_2_ − 1.0 (g/L). The main carbon source is glucose purity 98% (“Servicebio”, Wuhan, Hubei, China). The pH of the culture medium is maintained at 7.0–7.2 by adding the KOH titrant (“Sigma”, St. Louis, MO, USA); pH is measured using a Professional Meter PP-15 (“Sartorius”, Göttingen, Germany). For the synthesis of copolymers with various inclusions of the monomers 3-hydroxyvalerate, 4-hydroxybutyrate, and 3-hydroxyhexanoate, the precursors of these monomers were added to the medium: respectively, valerate, γ-butyrolactone, and hexanoate (“Sigma”, St. Louis, MO, USA). The number of additives of precursors varies from 1 to 3 to ensure the inclusion of the desired monomers in copolymers at various. The procedure for the synthesis of PHAs of various compositions, research methods, and properties is described in detail in [51].

Methods of extraction and the study of the composition and chemical purity of the samples obtained are described in detail in [51,52,53].

### 2.2. Production of Polymer Films

Thin polymer films have been obtained by casting a 2% polymer solution in dichloromethane onto previously degreased Teflon-coated templates, after which the films are placed in a dust-free laminar box (“Labconco”, Kansas City, MO, USA) until the solvent completely evaporates. The thickness of the films is measured with a LEGIONER EDM-25-0.001 electronic digital micrometer (“Legioner”, Shanghai, China). The film thickness is 298.94 ± 12.79 μm.

### 2.3. Laser Treatment Modes

Laser modification of thin polymer films is carried out by the method of moderate uniform surface irradiation using a LaserPro Explorer II CO_2_ laser (Coherent, Santa Clara, CA, USA) with the following characteristics: wavelength 10.6 μm, maximum power 30 W, maximum speed 2 m/s, maximum resolution 1000 dpi, SeZn lens (“LASER RESEARCH OPTICS”, Providence, RI, USA), F = 2’. To study the effect of laser modification modes, the power, speed, and wave continuity have been varied.

**Figure 1:**
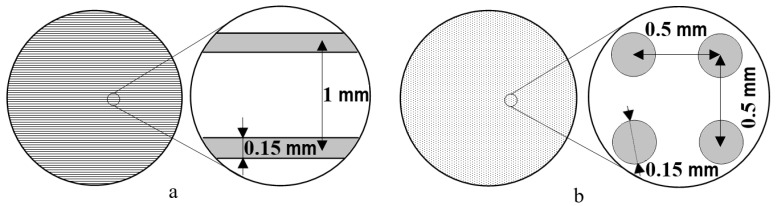
Graphic scheme of laser processing: (**a**)—continuous and (**b**)—quasi-pulse laser irradiation.

First mode (Figure 1a): surface modification is carried out in the continuous melting mode with a distance between the vector lines of laser passage of 1 mm: power 3 W, specific power 20,000 W/m^2^, speed 2 m/s, beam diameter before the focusing lens 2.5 mm, after the focusing lens 0.15 mm.

Second mode (Figure 1b): surface modification is carried out in a quasi-pulse mode, with a distance between the raster dots of pulses of 0.5 mm: power 15 W, specific power 100,000 W/m^2^, speed 1 m/s, beam diameter before the focusing lens 2.5 mm, after the focusing lens −0.15 mm.

### 2.4. A Study of Polymer Films Surface

The porosity and surface structure of thin films have been studied using a high-resolution scanning electron microscope FE-SEM S-5500 (Hitachi, Tokyo, Japan). All samples are sputtered with platinum (at 25 mA for 60 s) using an EM ACE200 (Leica, Vienna, Austria). Surface porosity is determined from micro images using the Digital Image Analysis Software Package (a free and open source software package for scientific analysis, editing, and bitmap processing), Image J v1.53k. The hydrophobic–hydrophilic balance (contact angles wetting) of the surface is measured on a Drop Shape Analyzer DSA-25E (Krüss, Germany) using the DSA-4 program for Windows. The free surface energy and its polar and dispersion components (mN/m) are calculated by the method of Owens, Wendt, Rabel and Kaelble.

The arithmetic mean roughness (Sa), root mean square roughness (Sq), and peak-to-valley height (Sz), which includes the entire range of values, that is, the total difference between the minimum and maximum of the profile irregularities, are determined using atomic force microscopy (AFM) in the semi-contact mode DPN 5000 (NanoInk, Skokie, IL, USA) according to the usual equations [54]. AFM data processing and statistical analysis of images are performed using the free software Gwyddion (2.51).

### 2.5. Cell Cultivation

Disks from PHAs film with a diameter of 10 mm have been obtained by cutting. Sterilization is carried out in a Sterrad NX sterilization system (Johnson&Johnson, New Brunswick, NJ, USA) with H_2_O_2_ plasma.

The adhesiveness and proliferative potential of the investigated thin polymer films are studied in a culture of mouse fibroblasts NIH 3T3 (ATCC, Manassas, VA, USA), which have been seeded on films at a concentration of 5·10^3^ cells/cm^2^ and placed in 24-well plates. Cultivation is carried out according to the standard method in DMEM medium with the addition of 10% fetal calf serum and an antibiotic solution (streptomycin 100 μg/mL, penicillin 100 IU/mL) (Gibco, Invitrogen, Waltham, MA, USA) in an atmosphere of 5% CO_2_ at a temperature of 37 °C in a CO_2_ incubator (New Brunswick scientific, Edison, NJ, USA).

Fluorescent staining of the cytoplasm and nuclear DNA is performed using fluorescein-conjugated phalloidin (FITC) and DAPI, respectively (Sigma-Aldrich, St. Louis, MO, USA), to assess the morphology of the formed cell layers. The viability of cultured fibroblasts is assessed in the MTT test using 3-(4,5-dimethylthiazol-2-yl)-2,5-diphenyltetrazolium bromide (Sigma). The test is based on the ability of live cell dehydrogenases to reduce the MTT reagent (“Sigma”, St. Louis, MO, USA) to formazan. The MTT solution (10 µL) is added to the wells with cells cultured on the surface of thin polymer films and to the wells with controls, and the volume is adjusted to 200 µL with the prepared nutrient medium. After 4 h of incubation, the films are transferred to clean plates and filled with DMSO to dissolve the formed MTT-formazan crystals. After 30 min, the supernatant is transferred to a 96-well plate and the optical density is measured at a wavelength of 540 nm using a Bio-Rad 680 microplate reader (Bio-Rad LABORATORIES Inc., USA). The number of cells is determined from the calibration graph.

### 2.6. Statistics

Statistical analysis of the results is performed by conventional methods using the standard software package of Microsoft Excel. Each experiment has been performed in triplicate. Arithmetic means and standard deviations have been found. The statistical significance of results has been determined using Student’s t-test (significance level: *p* ≤ 0.05). The impact scores of the two laser processing modes are linearly normalized, and the corresponding radar charts have been generated based on the normalized evaluation score values.

## 3. Results and Discussion

Laser processing of films obtained from PHAs (Table 1) is carried out using an electric discharge gas CO_2_ laser. The choice of laser and mode is because this type of laser is the most powerful and common among long-range lasers. The radiation is mainly generated at a wavelength of 10.6 µm. The efficiency of such lasers exceeds 10%, and they can generate high-quality radiation with a power of several kilowatts [37]. CO_2_ lasers are widely used for various material processing—cutting, welding, and engraving, as well as in laser surgery.

The choice of processing modes is based on the fact that the main processes of modifying the surface of polymeric materials under the action of laser radiation are evaporation and melting. The process characterizing the formation of cavities in the forms of stripes and grooves, holes, etc., suggests that the depression grows in depth due to the evaporation of the material, and in diameter due to the melting of the walls and the displacement of the liquid phase by excess vapor pressure. The variable parameters are two processing modes: in the focused mode, continuous wave processing is carried out linearly (line by line)—the melting mode, and in the quasi-pulse mode, raster engraving (dot) is used—the evaporation mode.

### 3.1. Modification of Polymer Film Surfaces in the Continuous Wave Mode

In the process of research of films obtained from various types of PHAs with different properties (Table 1), it is necessary to reveal differences in the response of surfaces to laser irradiation depending on the set and ratio of monomers. Previously, it has been found that the original (untreated) films obtained from PHAs of various compositions, with the same thickness (298.94 ± 12.79 μm), have significant differences in morphology and surface characteristics [51],—samples obtained from copolymers have increased roughness values, reduced values of the contact angle of wetting with water, and greater porosity compared to homogeneous P(3HB).

**Figure 2:**
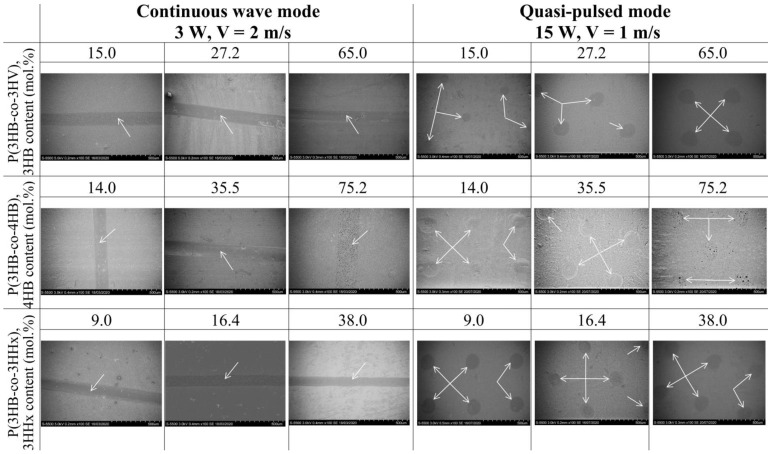
SEM images of surfaces of the laser-treated films prepared from PHAs with different compositions. Bar = 500 µm. Arrows indicate ablated zones.

Figure 2 shows SEM images of polymer films treated continuously. It can be seen that smoothed (melted) grooves have formed on the surface of the films at the site of the laser beam impact. This is due to the fact that in this mode during laser processing, a polymer melt is formed, above which an increased vapor pressure is formed, which prevents the removal of the melt. Therefore, as a result of subsequent cooling in the place of the melt, melted zones are formed in the form of grooves.

The temperature characteristics of polymers, melting point (T_melt_) and thermal degradation temperature (T_degr_), determine the conditions for obtaining and/or processing polymer products from melts and their properties. As shown earlier, the family of PHAs samples under study is characterized by some differences in temperature characteristics [51]. So, P(3HB) has a softening point of about 110 °C and a crystallization temperature (T_c_) between 85 and 110 °C, and the range of melting and thermal degradation, respectively, is 170–182 and 280–300 °C (Table 1). All copolymer samples, while maintaining the gap between T_melt_ and T_degr_, have lower melting temperatures. Thus, P(3HB-co-3HV) copolymers have two peaks in the melting region and the widest melting ranges from 107 to 168 °C, while samples with a high content of 3HV monomers have a lower T_melt_, which decreases from 163.9 to 130 °C for the content of monomers 3HV 65 mol.%. In P(3HB-co-4HB) copolymers, as the 4HB monomers increase, thermal analysis shows two melting peaks and two crystallization peaks. This type of copolymers has the lowest glass transition temperatures, which increase with increasing 4HB monomers content. For copolymers containing 3HHx monomers, the melting temperature ranges from 168 to 170 °C, with melting peaks being sharper than those of other copolymers and no doubling of melting peaks observed; the glass transition temperature ranges from −1.6 to −0.2 °C, decreasing with the increasing of 3HHx monomers content.

Differences in the temperature properties of the studied film samples influence the character of PHAs melting under the action of laser radiation, the formation of melted zones on the surface of the films, and their sizes. This is reflected in the width of the melted grooves and the total area of the modified zones (Table 2). Thus, for films obtained from the most heat-resistant and highly crystalline P(3HB), which has the highest melting and thermal degradation temperatures, the width of melted grooves is the narrowest (115.75 µm), and the total area of the modified surface is the lowest (11.51% of the total area).

Almost all samples of copolymer films that have different temperature characteristics, including melting, glass transition, and crystallization temperatures, show an increase in the modified area formed as a result of laser treatment. The exception is the samples from the copolymer P(3HB-co-4HB) (Table 2). Thus, for film samples obtained from P(3HB-co-3HV) as a result of more active melting under the action of continuous laser radiation, an increase in the width of the formed melted grooves is noted, but without a direct relationship with the value of 3HV monomers in the copolymer in the studied range of values. Thus, at the content of 3HV monomers 15.0 and 27.2 mol.%, the width of melted grooves increases to 132.7 and 140.2 µm, and the modified area is 13.1 and 13.6%. However, at the maximum content of 3HV monomers in the copolymer (65.0 mol.%), the groove width is lower, as is the total modified area. A similar effect of continuous wave mode has been found in the other two types of copolymer films examined. To explain this contradictory effect, special studies of the kinetics of polymer crystallization in the cooling process after laser treatment are needed, with the registration of the process of the formation and size of the formed spherulites.

Even larger melting zones and total modified surface area after continuous laser treatment have been recorded for films made from P(3HB-co-4HB) copolymers. For film samples made from copolymers containing 14.0 and 35.5 mol.% 4HB monomers, the total modified area is 13.4 and 15.9%, and the width of melted grooves increases to 135.6 and 163.3 µm, respectively. However, at the highest content of monomers, 4HB (75.5 mol.%), the changes under the action of laser radiation are least pronounced: the width of the melted grooves is 105.5 µm, and the area of the modified surface is 10.4%. For this type of films, laser processing causes additional pore formation in the zones of action of the laser beam. Numerous pores are formed on the initially most porous P(3HB-co-4HB) films after laser treatment, and most of them are large (3.0–3.5 μm) compared with other samples in the investigation (Figure 2).

The mode of continuous laser radiation similarly affects the modification of the surface of films made from P(3HB-co-4HHx) copolymers, also without a clear relationship with the content of 3HHx monomers. Thus, when the content of these monomers in the copolymer is 9.0 and 16.4 mol.%. the width of the stripes is maximum 145.5 and 152.5 µm, respectively, and the total modified area is 14.1 and 14.9%. On the contrary, at the maximum content of 3HHx monomers (38.0 mol.%), the width of the grooves is smaller (125.1 µm) and the total value of the modified area is also lower (12.3%), but this, nevertheless, is higher than in the case of laser modification of films from homopolymer P(3HB).

An analysis of the literature reveals the presence of similar data on the effect of laser treatment on the change in the surface morphology of polymer film products from various materials. It has been shown that the treatment of P(3HB) products with a nitrogen-blanketed CO_2_-laser results in the formation of specific zones in the form of grooves 60 to 100 μm deep with a structure that differs from the original one [43]. Using the example of films obtained from the poly(3-hydroxyoctanoate-co-3-hydroxydecanoate) copolymer (a member of the PHAs family) and mixtures of this copolymer with the P(3HB) homopolymer, which initially differ in thermal and mechanical properties, it is shown that, upon processing with the Picosecond Laser Ablation, grooves and cavities are formed on the surface of the films, the depth and size of which depend on the composition of the polymer material and the processing mode [55]. During the treatment of polylactide films with the CO_2_ laser, depressions in the form of grooves and ridges protruding above the surface, repeating at a distance of 40 to 60 μm, are formed on the surface [56]. The authors of [57] have observed folds and depressions during processing with an excimer laser. Thus, laser processing allows one to modify the surface of polymer products and cause significant changes in its morphology, affecting adhesive and other properties.

The surface roughness of polymer products for biomedicine is an important parameter characterizing the reactivity of the surface, which, at the nanometer level, affects protein adsorption, cell adhesion, proliferation, and the synthesis of specialized structural proteins and extracellular matrix proteins. 

**Figure 3:**
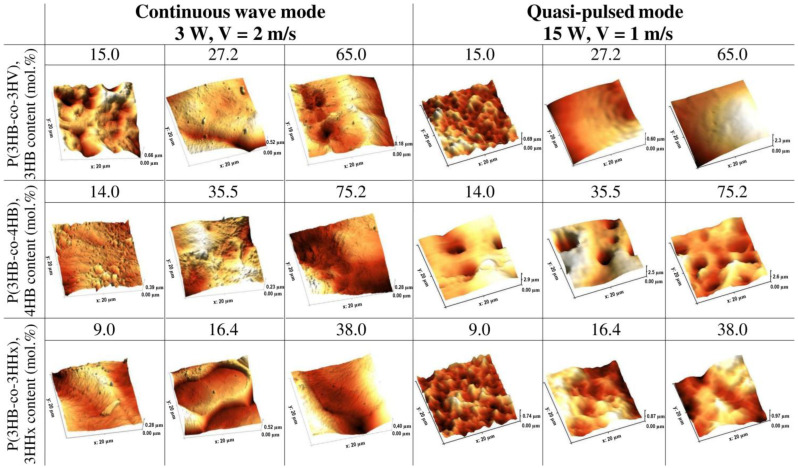
AFM images of surfaces of the laser-treated films prepared from PHAs with different compositions.

Figure 2 and Figure 3 and Table 3 show the results of the influence of laser modification on the surface roughness values of thin polymer films obtained from PHAs of different chemical compositions.

Depending on the chemical composition of PHAs, the reactions of the surfaces of 10 studied samples of polymer films in response to continuous laser irradiation differ, which is consistent with the previously obtained results upon irradiation of films from four types of PHAs [50].

**Figure 4:**
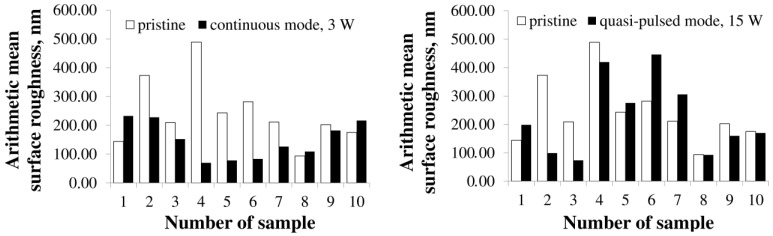
The effect of the composition of 2-component PHAs on the value of the arithmetic mean (Sa) after laser treatment in continuous and quasi-pulse modes (numbering—Table 1): 1—P(3HB); 2–4—P(3HB-co-3HV); 5–7—P(3HB-co-4HB); 8–10—P(3HB-co-3HHx).

Irradiation of P(3HB) films causes an increase in all studied roughness indices: arithmetic mean surface roughness (Sa) up to 232 nm and root mean square roughness (Sq) up to 282 nm, as well as the integrated peak-to-valley height (Sz) up to 1,692 nm compared to the original unprocessed films, in which all values are lower, respectively 154, 180, and 1,255 nm. Changes in the roughness of copolymer samples manifest themselves in different ways and depend not only on the PHAs’ composition, but also on the ratio of monomers (Figure 4; Table 3). For example, continuous irradiation changes the surface roughness of films made of P(3HB-co-3HV) copolymers differently depending on the content of 3HV monomers. At the lowest content of these monomers (15 mol.%), all roughness indices slightly increase. On the contrary, with an increase in the content of 3HV monomers in the copolymer, all indicators decrease, which is especially significant, almost three times as compared with untreated films, at 65 mol.% 3HV. All samples from another type of PHAs, P(3HB-co-4HB) copolymers, respond to continuous laser radiation in the same way. The decrease in the arithmetic mean roughness index is the most significant, down to 78 and 83 nm, for samples with 4HB monomer contents of 14.0 and 35.5 mol.%. These are the lowest values of the entire series of studied samples. At a higher monomer content of 4HB (75.2 mol.%), the Sa value also decreases, but to a lesser extent, 126 nm. Film samples obtained from P(3HB-co-3HHx) copolymers also react differently to continuous radiation depending on the content of 3HHx monomers. The roughness of the films obtained from the copolymer with the highest monomer content (38.0 mol.%) changes a little; the Sa, Sq, and Sz values are close to those of the untreated films. With a decrease in the content of 3HHx monomers to 16.4 mol.%, the Sa and Sq indices are also close to the untreated films, but the profile height (Sz) increases to 1,457 nm. And, finally, at the minimum content of 3HHx monomers in the copolymer (9.0 mol.%), all roughness indicators decrease after irradiation of the films (Table 3).

No less important are the energy characteristics of the surface of polymer products, which characterize the hydrophilic–hydrophobic balance and affect the adhesion and viability of cells. An indicator of this ratio is the value of the contact angle of wetting the surface with liquids. It is known that films made from PHAs of various chemical compositions have differences in surface characteristics, including the contact angle of wetting with liquids. It has been shown that the values of the contact angle for P(3HB) films are the highest on the order of 90° and more [51], while for copolymer films this index is lower by 10–50°, depending on the set of monomers.

The value of the contact angle for the films obtained from the P(3HB) homopolymer after continuous laser treatment decreases to 80.5° from the initial value of 92.1°. Copolymer films of various compositions, which initially have low contact angles, the minimum (41.9°) for P(3HB-co-3HHx) films and the maximum (82.4°) for P(3HB-co-4HHx) films, respond to laser treatment differently (Figure 4).

The value of the angle significantly increases in films with a low (9.0 mol.%) content of 3HV monomers by more than 20°, to a level comparable to that of P(3HB) films. In two other samples, with an average and the highest content of 3HB monomers, the observed minor changes are not significant. A significant decrease in the angle, from 82.2° to 69.5°, is recorded for films made of the P(3HB-co-4HB) copolymer with the lowest content of 4HB monomers (14.0 mol.%). The angle of the films with a higher content of monomers (4HB) does not change significantly in response to laser treatment, remaining at the level of the original samples. Another picture has been obtained by irradiating films from the P(3HB-co-3HHx) copolymer. At the average and highest content of 3HHx monomers (16.4 and 38.0 mol.%), irradiation leads to a significant increase in the contact angle, by 71 and 43%, respectively. However, the angle does not change for the films with the lowest content of 3HHx monomers. Thus, laser processing in the continuous mode of radiation, in addition to changing the roughness indices, causes multidirectional changes in the contact angle of the film surface depending on the set of monomers in copolymer PHAs, as well as on the inclusion of second monomers other than 3HB.

Comparison of the obtained results with publications shows that films made from the P(3HB) homopolymer are most often subjected to laser treatment. Dr. Lootz et al. have shown that the processing of P(3HB) products using a nitrogen-blanketed CO_2_-laser results in the formation of specific recessed structures of varying depth (from 60 to 100 µm) [43]. It is shown that the use of different modes of irradiation of P(3HB) films using a CO_2_ laser makes it possible to obtain a family of samples with various surface changes, from the formation of slight roughness to the formation of through perforations; the resulting films had reduced values of the contact angle [44]. In [45], the effects of processing flexible P(3HB) films with two CO_2_ lasers (LaserPro Explorer and LaserPro Spirit) have been studied when changing the processing power and speed in focused and defocused modes. The authors of this article demonstrate a decrease in the contact angle after modification in focused or defocused modes at a processing speed of 0.8 m/s and 1.8 m/s and a power of 9 W and 12 W, respectively. In [47], Michaljaniová et al. have modified P(3HB) and polylactide films of homogeneous poly-3-hydroxybutyrate and polylactide with KrF and ArF excimer lasers. It is shown that, under the same processing conditions, the ArF laser causes more significant changes in the surface structure. Modification of poly(3-hydroxybutyrate) with a KrF laser causes an opposite effect on the surface morphology compared to polylactide. At a low energy of the laser beam (up to 15 mJ/cm^2^), there is no effect on the surface of the material; however, as the laser radiation flux density increases, the surface roughness increases rapidly. Increasing the pulse frequency causes a significant increase in the roughness of poly-3-hydroxybutyrate. In [46], Çatıker et al. have modified films of poly-3-hydroxybutyrate, polylactide, and poly(methyl methacrylate, and polyurethane) with an excimer KrF laser, with the help of which cavities and ordered perforations have been formed on the films.

Regarding the laser modification of the surface of films made of P(3HB-co-3HV) copolymers, several works are known in which samples with a low (about 10 mol.%) content of 3HV monomers are used. The formation of micropores 150 × 100 µm in size, 11.7 × 10^3^ mm^2^ in area, and up to 200 µm apart from each other is observed on samples of this copolymer with a 3HV monomer content of 11 mol.% after Nd:YAG laser irradiation [48]. On films of the same copolymer with a similar content of 3HV monomers, processed using ultraviolet laser ablation, the fourth harmonic of a Nd:YAG laser, pores with a diameter of 100 µm are also formed, which are evenly spaced at intervals of 200 µm [49]. Films obtained with a 3HV monomer content of 8 mol.% are processed by a KrF laser and also processing with Ar^+^ plasma. This causes a decrease in the contact angles from 60–68° to 40–54° and significantly increases the surface roughness [58].

The results of studying the consequences of laser processing of polymer products made from copolymers P(3HB-co-4HB) and P(3HB-co-3HHx) in the available literature have not been found. At the same time, the effect of laser processing on the change in the morphology and surface roughness is shown on other polymeric materials. Thus, on the surface of films obtained from carbon-coated polyethylene, the wettability and roughness indices increase after laser treatment [59]. The modified surface of polylactide films after CO_2_ laser treatment has changes in roughness and wettability; Femtosecond laser modification of thin chitosan films increases the surface roughness from 0.5 to almost 3 μm [60,61].

The revealed opposite changes in the angle in response to laser irradiation in the studied film samples obtained from PHAs of various chemical compositions, apparently, can be legitimately associated with differences in their properties, particularly the degree of crystallinity (Table 1). As is known, the semi-crystalline polymers, which include PHAs, have both crystalline and amorphous regions. Crystalline regions provide the polymer material with high hardness and thermal stability, and the amorphous zones give the material a certain elasticity and impact resistance. The properties of the semi-crystalline polymers depend on the degree of crystallinity, the size and distribution of the crystals, and the properties of the interface between the amorphous and crystalline regions [62].

The obtained results of changes in the roughness of PHAs polymer films of various chemical compositions and the value of the contact angle of wetting with water after continuous laser irradiation do not correspond to the established ideas and models of Cassie Baxter and Wenzel, which characterize changes in the wettability of surfaces in relation to roughness. Thus, using the Cassie-Baxter model, amorphization and an increase in the hydrophobicity of the surface are explained as the polar components of the free surface energy increase and the roughness indices change. In this case, the increase in hydrophobicity is due to the formation of a Cassie−Baxter state in which the liquid does not penetrate into the hollows of the corrugated surface and, consequently, faces a composite interface consisting of both solid and vapor. In this case, the increase in hydrophobicity is due to the formation of a Cassie−Baxter state in which the liquid does not penetrate into the hollows of the corrugated surface and, consequently, faces a composite interface consisting of both solid and vapor [63,64,65]. The increase in surface hydrophilicity corresponds to the Wenzel model, which describes a uniform wetting regime and predicts a change in surface wettability due to an increase in surface roughness [66].

It should be noted that the development of the theoretical concepts of the models of Cassie–Baxter and Wenzel reveals the presence, in a number of cases, of limitations for their application. So, A. Giacomello and colleagues have used atomistic simulations aimed at computing the free energy of the stable and metastable states of the system as well as the intermediate states separating them, and have found that the usual description in terms of the Cassie−Baxter and Wenzel states is insufficient, as the system presents two states of the Cassie−Baxter type. These states are characterized by different curvatures of the meniscus. The measured free energy barrier separating the Cassie−Baxter from the Wenzel state (and vice versa) largely exceeds the thermal energy, attesting the existence of Cassie−Baxter/Wenzel metastabilities. Finally, it has been found that the Cassie−Baxter/Wenzel transition follows an asymmetric path, with the formation of a liquid finger on one side of the groove and a vapor bubble on the opposite side [67].

It should be emphasized that the wettability of the surface, which changes as a result of the formation of laser-induced periodic surface structures (LIPSS), depends not only on the microrelief and roughness indices, but also on other factors.

As shown by A. B. D. Cassie and S. Baxter [63], in the case of porous surfaces, this specificity should be taken into account when determining the magnitude of the angle during the application of liquid droplets to the surface. In our case, the porosity of the films, including the number and size of pores as well as their total area, obtained from PHAs of various chemical compositions, differs, and this applies both to the original untreated films as well as those treated with laser radiation. This fact may influence the correctness of the obtained values of the contact angles, which are measured by the sessile drop method using water as a reference liquid.

Busscher et al. [68] have determined the advancing and receding contact angles for five different fluids on 12 commercial polymers after various surface roughening procedures, and show that roughness tends to increase in contact angles if the contact angle on a smooth surface exceeds 86°, then how the contact angle decreases if the angle on a smooth surface is less than 60°. Therefore, for contact angles in the range of 60° to 86°, the surface roughness does not affect the measured angles. These results show that the trends predicted by the Wenzel equation are consistent. It has also been found that the type of reference liquid can affect the behavior of the drop on the surface and the value of contact angles [69]. The authors of this work have used the sessile drop technique to measure the contact angles of the laser-treated surfaces using water, glycerol, and paraffin oil, and have found differences in the angles, which, as it turns out, depend not only on the irradiation mode, but also on the type of test liquid. Therefore, we believe that it is necessary to take this into account and use a wider set of reference liquids to measure angles in future work.

The change in the wettability of surfaces is also influenced by the regime itself and the conditions of the medium during laser irradiation. Changes in the input parameters (for example, laser flux and scan area) lead to a change in the surface and the magnitude of the angles. Scaffolds that have been treated in air or oxygen show an increased concentration of atomic oxygen and also ablation of the material. Oxygen concentrations tend to be higher and contact angles are smaller for surfaces where ablation is more pronounced. This effect is independent of the fact that the ablation is caused by higher laser power, reduced scan space, or shorter defocus distance [70]. It is shown that the wettability of polyethylene (PE) and polyimide (PI) films, which is characterized by the contact angles, increases significantly after exposure to an excimer laser in an air atmosphere. A subsequent study of the irradiated layers by X-ray photoelectron spectroscopy (XPS) shows that a decrease in the angle value is associated with an increase in the oxygen concentration on the modified surface; at the same time, the characteristics of the surface of the film do not change significantly under the influence of ultraviolet radiation, and the aged PE and PI films exposed to ultraviolet radiation show a significant decrease in wettability [71]. This is an indicator of the absence, in some cases, of a direct relationship between wettability and surface roughness.

There are works that show the influence of the chemical composition of the material on the consequences of laser processing. For example, when F_2_ laser processing films are obtained from various polymers (PE), polypropylene (PP), polytetrafluoroethylene (PTFE), polystyrene (PS), and polyethylene terephthalate (PET), it is found that the chemical composition affects the ablation and degradation of the surface layer of the polymer and, consequently, the influence on the contact angle of the liquid with the surface and the value of the contact angle [72]. On the contrary, when polylactide films are irradiated with a CO_2_ laser against the background of a change in surface roughness, no change in wettability is detected, which indicates the absence of the formation of polar functional groups [73].

In general, these published data, as well as the complexity and ambiguity of the results obtained during laser processing of PHA films with different chemical compositions and properties, indicate the need for special theoretical and experimental studies to understand the patterns of changes in the surface structure of polymer films obtained from these semi-crystalline and degradable polymers.

### 3.2. Modification of Polymer Film Surfaces in the Quasi-Pulsed Mode

The second studied mode of processing polymer films from PHAs of various compositions (Table 1) is quasi-impulse. It should be noted that not a true pulsed mode is used, but a quasi-pulsed mode using an optical system of mirrors, which provides interruption of the constant laser beam. This mode is performed by the raster method (dots) in the focused mode at a processing power of 15.0 W. In contrast to the continuous mode, in the quasi-pulse mode of processing, the temperature of the material in the irradiation zone is higher than the boiling point; therefore, its removal in the surface of the material being processed occurs in the form of a vapor-drop phase. The processing power is taken based on preliminary studies, which show that at a power of less than 13 W, material ablation does not occur; above 15 W, through perforations are formed, in the presence of which it is not possible to conduct correct studies of the properties of the surface of irradiated films [50].

As a result of the processing of films in the quasi-pulse mode, SEM images (Figure 2) show the formation of defects in the form of depressions (holes) on the surface of all types of films at the site of exposure to the laser beam. When P(3HB) films are irradiated with a power of 15.0 W, the diameter of the holes formed on the surface is 151.25 μm and the total modified area is 7.24% (Table 2). The diameter of most holes on the surface of copolymer films is generally higher and somewhat different for samples of different compositions. Similar to the effect of the continuous wave mode, the absence of the effect of increasing the diameter of the holes and their total area occurs during the treatment of films from P(3HB-co-3HV) and P(3HB-co-3HHx) with close contents of 3HV and 3HHx monomers (respectively, 15.0 and 16.4 mol.%) (Table 2).The diameter of the wells in three samples of films obtained from P(3HB-co-4HB) copolymers after treatment in a quasi-pulse mode, regardless of the content of 4HB monomers (14.0–75.2 mol.%), is close and is in the range 167.6–174.8 µm, as well as the total area of the modified regions (8.7–9.0%) processing mode. Film samples of this composition are characterized by the formation of an additional number of large pores (with a diameter of up to 3.0–3.5 μm) at the site of laser beam impact, which is similar to the effect of continuous processing.

The hole sizes (192.0–177.0 µm) and their total area (11.3–9.9%) in two samples obtained from P(3HB-co-3HV) copolymers with an average and the highest content of 3HV monomers are higher than for films from P(3HB), but comparable. That is, they do not depend on the ratio of monomers in this type of copolymer. A similar effect of the absence of influence of the content of second monomers on the sizes of formed holes after laser irradiation in the quasi-pulse mode is typical for films obtained from another type of PHAs—copolymers P(3HV-co-3HHx) with the lowest (9.0 mol.%) and the highest (38.0 mol.%) content of 3HH monomers. The diameter of the wells in these two samples and their total area are the same, about 160 µm and 8.3–8.3%. It should also be noted that the dimensions of the holes and their total area in copolymer samples from PHAs with an average content of second monomers (close to 30 mol.%) are similar to the previously obtained results in the quasi-pulse mode of processing copolymer films of a similar composition, but at a lower radiation power (13.5 W) [50].

The mode of quasi-pulse laser radiation, similarly to the continuous processing mode, has an impact on the surface roughness of polymer films (Table 3, Figure 3 and Figure 4). The surface roughness indices of the films obtained from P(3HB) increase after laser treatment. The quantitative values of Sa and Sq increase after treatment by 78 and 65 μm, respectively; that is, by 25–30% compared with the untreated films of this type.

The effect of quasi-pulse radiation on the surface roughness indices of copolymer films is ambiguous and depends not only on the chemical composition of the copolymers and on the type of the second monomer, but also on its content in the copolymers. All roughness indices (Sa, Sq, and Sz) for films obtained from the P(3HB-co-3HV) copolymer with a low (15.0 mol.%) and medium (27.2 mol.%) content of 3HV monomers decrease by 3 or more times relative to untreated films, amounting to arithmetic mean and root mean square roughness of the order of 100 microns or less; the Sz values are also low, on the order of 600–690 µm. However, with an increase in the content of 4HV monomers to 65 mol.%, the roughness indices Sa, Sq, and Sz increase, respectively, to 419; 486, and 2,284 µm. In the continuous processing mode, the roughness indices of this sample, on the contrary, decrease significantly.

A change in the content of 4HB monomers in the P(3HB-co-4HB) copolymers has the same effect on the film surface roughness parameters. Thus, upon irradiation of the films, regardless of the monomers containing 4HB (14.0; 35.5; 75.2 mol.%) after the quasi-pulse treatment, the values of Sa increase, respectively, to 275, 442, and 5,153 µm; Sq—up to 442, 561, and 388 µm; Sz—up to 305, 388, and 2,612 microns, which is higher compared to untreated films (from 10–15 to 100% or more) and several times higher than all these indicators for films after processing in continuous mode, which has the opposite effect—causing a decrease in roughness.

For films obtained from the P(3HB-co-3HHx) copolymer, the effect of the quasi-pulse processing mode is generally comparable with the effect of the continuous processing mode; the quantitative indicators of roughness are basically similar. All measured roughness indices for film samples, regardless of the content of 3HHx monomers in the copolymer, are low and close to those for untreated films, except for the value of Sz, which decreases to 869 and 972 μm at a 3HHx content of 16.4 and 38.0 mol.%, which is inferior to the values of untreated samples and after continuous irradiation (1200–1600 µm).

Thus, the quasi-pulse mode of laser processing increases the roughness indices for all types of P(3HB-co-4HV) copolymer films and the P(3HB-co-3HV) sample with the highest content of 3HV monomers. In relation to the rest of the samples, the effect is generally the opposite, and the roughness indices decrease relative to the original films. The complexity and ambiguity of the influence of the composition of polymers on the surface roughness indices of PHAs films has been noted in a number of works. For example, it has been shown in [74] that the inclusion of 3HV monomers increases the surface roughness of copolymer films. Thus, atomic force microscopic analysis shows that the surface roughness values of all films from P(3HB-co-3HV) with 26 and 12 mol.% HV are 92.5 and 290.8 nm, and with the addition of polyethylene glycol, it increases to 588.8 nm [75]. At the same time, there is evidence that an increase in the content of 4HB monomers in the P(3HB-co-4HB) copolymer is accompanied by a decrease in the surface roughness of the films, which become smoother [76,77].

**Figure 5:**
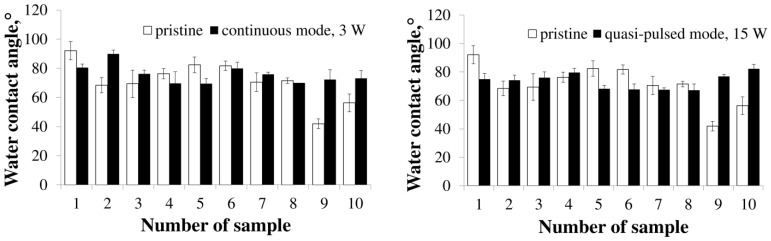
The effect of the composition of 2-component PHAs on the value of the contact angle after laser treatment in continuous and quasi-pulse modes (numbering—Table 1): 1—P(3HB); 2–4—P(3HB-co-3HV); 5–7—P(3HB-co-4HB); 8–10—P(3HB-co-3HHx).

The results of the effect of quasi-pulse laser treatment on the value of the contact angle are shown in Figure 5. As it is noted earlier [50], the given contact angle values are the averaged measurement data not only of the modified areas (formed dimples), but also of the untreated interaholes space. This is due to the fact that the size of the droplets used to measure the contact angles significantly exceeds the size of the wells, so the area of the liquid drop applied for measurement covers 4–5 wells with the space between them.

Similar to the effect of the continuous treatment mode, as a result of quasi-pulse treatment of the surface of films made from the P(3HB) homopolymer, the value of the contact angle significantly decreases, amounting to 74.9°, relative to 92° for the initial films. The angle of the initial copolymer films is lower than that of the homopolymer (it is noted above). A significant change in the angle value after irradiation of films from all types of P(3HB-co-3HV) copolymers is not observed; values remain close to those of the untreated films. Surface hydrophilization takes place during irradiation of films obtained from the P(3HB-co-4HB) copolymer with a low (14 mol.%) and medium (35.5 mol.%) content of 4HB monomers, for which a significant decrease in the angle values is recorded (Figure 5). At the same time, an increase in the content of 4HB monomers to 75 mol.% does not affect this parameter. The opposite effect, namely, a pronounced increase in the angle to 77 and 82° relative to the initial 42 and 56°, is obtained for two samples of films from the copolymer P(3HB-co-4HHx) with a content of 3HHx monomers of 16.4 and 38.0 mol.%. This is similar to the effect of continuous radiation for these samples. Thus, with the exception of P(3HB-co-3HHx) films, the quasi-pulse treatment reduces the contact angle meaning or does not significantly change the values. In general, the majority of films treated in this mode have a lower angle, which indicates their greater degree of hydrophilicity.

Differences in the degree of crystallinity in the studied samples of films obtained from PHAs of various compositions, most likely, affect the formation of the melt and the evaporation processes in the zone of exposure to the laser beam, as well as the subsequent crystallization of the material as it cools. These processes could cause differences in surface formation in the formation of LIPSS [78,79]. In this case, the process of LIPSS formation depends both on the nature of the irradiation regime and on the structural features of the irradiated polymer material.

In principle, taking into account the larger size of the obtained structures both in terms of period and depth, these results may be consistent to some extent with the Cassie–Baxter model for explaining the increase in surface hydrophobicity after laser irradiation of individual film samples obtained from the P(3HB) homopolymer and P(3HB-co-3HHx) copolymers of these samples. According to this model, the contact angle can increase even if the contact angle of the liquid on the original (unmodified) surface is less than 90° [63].

As noted above, in the present work for the processing of polymer films, a quasi-pulse mode has been used using an optical system of mirrors, which interrupts continuous laser irradiation, which makes it difficult to discuss the results of colleagues who have used the true pulsed mode of laser irradiation. Thus, in a series of deep and fundamental works by colleagues from Spain, the regularities of the formation of LIPSS on the surface of various materials under various modes of pulsed irradiation with a pulse duration in the nanosecond, picosecond, and femtosecond ranges, as well as in the range of lengths waves from ultraviolet to infrared. At the same time, it has been found that, depending on the type of initial material and the degree of wettability of the surface of products made from it, as well as the mode of laser radiation, the consequences of laser processing can be different and lead both to the hydrophilization of the surface of the original samples, and, conversely, to an increase in their hydrophobicity [80,81,82,83].

Thus, this research team, through using the example of thin films of PET and PET reinforced with foamed graphite (EG) under laser irradiation with ultraviolet (265 nm) and near-infrared (795 nm) femtosecond laser pulses, shows that LIPSS forms in both materials, but by different mechanisms [69]. In some cases, laser processing leads to an increase in hydrophobicity; and in others, on the contrary, it causes a decrease in hydrophobicity and an increase in the hydrophilicity of surfaces. The initially hydrophilic surfaces become more hydrophilic after ultraviolet irradiation, while they evolve to become hydrophobic under near-infrared laser irradiation—for UV-irradiated surfaces, adhesion, determined by the colloidal probe technique, increases, while, for NIR irradiation, adhesion decreases. The authors have concluded that while the original surfaces have had a hydrophilic character before irradiation, upon laser irradiation in the UV, samples become more hydrophilic, which can be explained through Wenzel’s model. In contrast, upon irradiation with NIR, the surfaces acquire a hydrophobic state. Using the Cassie–Baxter model, it is possible to explain this behavior. The authors relate the differences in the effects of laser treatment to the differences in the properties of the materials under study and the different intensities of laser irradiation, summarizing that absorption on the outer polymer layer proceeds via nonlinear mechanisms, such as multiphoton absorption and ionization processes. These results explain possible multidirectional changes in the structure of the surface of polymeric materials, which are influenced by the characteristics of the studied and irradiated material, as well as by the laser radiation regimes.

In general, the changes in the structure and properties of the surface of the studied polymer films obtained from PHAs of various compositions, with different ratios of monomers and irradiated with two different laser treatment modes, obtained in different directions and very difficult to interpret, should be considered today as preliminary results requiring the carrying out of special and theoretical, as well as additional experimental, studies.

### 3.3. Biological Properties of Laser-Treated PHA Films of Various Compositions

**Figure 6:**
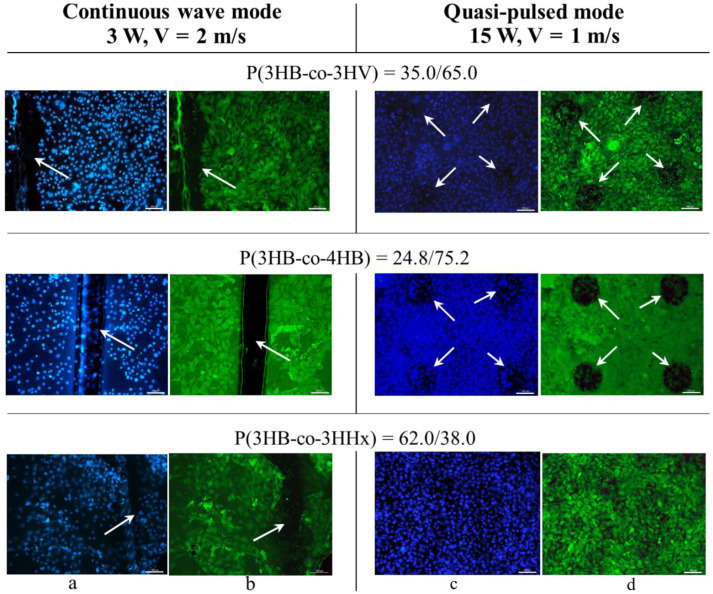
Photo of NIH 3T3 mouse fibroblasts stained with DAPI (**a**,**c**) and FITC (**b**,**d**) on PHAs polymer films of various compositions treated with continuous and quasi-pulse laser irradiation, bar = 100 µm.

**Figure 7:**
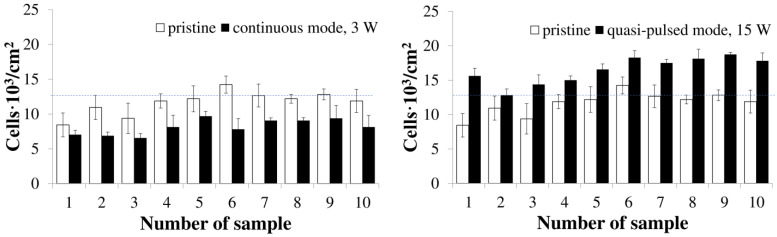
The number of viable NIH 3T3 mouse fibroblasts cultured on polymer films of various compositions treated with continuous and quasi-pulse laser irradiation compared to the original untreated films (2-day culture); dashed line—results in control—number of cells on polystyrene culture plates.

The studies have been performed in a culture of mouse fibroblasts of the NIH 3T3 line. The biocompatibility of PHA polymer films of various compositions, depending on the laser irradiation mode, has been assessed by the results of fibroblast staining with fluorescent dyes DAPI (marker of nuclear DNA) and phalloidin conjugated with fluorescein FITC (marker of cell cytoplasm) (Figure 6), as well as in the colorimetric MTT test (Figure 7). All film samples, regardless of the physicochemical properties and laser modification mode, do not adversely affect the functional properties of cultured fibroblasts. Despite this, the number of viable cells, according to the results of the MTT test, vary and depend both on the physicochemical properties and on the mode of laser modification.

The difficulty in identifying the effects of laser treatment itself on cell adherence and proliferation lies in the fact that various PHAs compositions also affect cellular processes on the sample’s surface. In our previous study [51] in a culture of NIH 3T3 mouse fibroblasts using fluorescent staining with DAPI and a colorimetric test for assessing the metabolic activity of cells, MTT, it has been shown that the most copolymer films containing 3-hydroxyhexanoate and 4-hydroxybutyrate monomers, which have the lowest degrees of crystallinity, are favorable for cell growth.

It should be noted that a change in the cellular response can be associated with even minor changes in the surface profile at the nano and micrometer levels, which mediates the processes of adhesion and further proliferation of cells on the surface. However, the produced effect is not universal, since morphologically different types of cells differ in their sensitivity to changes in surface topography, which requires additional experiments on mammalian cell cultures of various origins in vitro.

The results show that the laser treatment mode has a different effect on the number of fibroblasts cultivated on polymer films of different composition. As an example, Figure 6 shows photographs of fibroblasts cultivated on films obtained from PHAs of various compositions and stained with fluorescent dyes. First of all, attention is drawn to the revealed fact of a reduced or almost complete absence of cells on the melted grooves formed on the surface of the films after continuous laser radiation (Figure 6). At the same time, after the quasi-pulse mode on the modified surface of the films, which are less melted areas in the form of holes (the total area of which is less than 10% of the area of the films), cells are present. It can also be seen that the number of cells on the films obtained from PHAs of various compositions differ.

Due to the fact that the total modified area, depending on the processing mode, differs significantly and is significantly higher for films after continuous processing (from 12 to 15% of the total area of films) compared to the quasi-impulse mode (8–9%), this significantly affects the number of viable cells, determined in the MTT test (Figure 7).

It should be noted that, regardless of the laser treatment mode and the PHAs composition used to obtain the films, no negative effect on the development of fibroblasts during their direct contact with the surfaces of all the studied samples, including all types of initial, untreated films, as well as both groups of irradiated films, has been detected. The number of viable metabolically active cells in the control (polystyrene) is lower to the indicators in all variants of the studied PHAs films (Figure 7).

PHAs films processed in the quasi-pulse mode are the most favorable as cell carriers. The number of fibroblasts on films of this type of all studied PHAs compositions is higher than the control (polystyrene) and depends very significantly on the composition of PHAs. Thus, on P(3HB) films, the number of fibroblasts is 85% higher than in the control, and even more significant (by 122%) is the number of cells on films irradiated in a continuous mode. The number of cells on films of P(3HB-co-3HV) copolymers is comparable with P(3HB), and is inferior to the values obtained on all films of other types of PHAs copolymers. At the same time, the lowest number of cells in the MTT test is recorded on films with the lowest content (15 mol.%) of 3HV monomers (12.8·10^3^/cm^2^). When analyzing the result of the MTT test when growing fibroblasts on films from P(3HB-co-4HB), a higher number (18.28·10^3^/cm^2^ and 17.5·10^3^/cm^2^) is obtained on films obtained from the polymer of this type with the highest content of 4HB monomers, respectively 35.5 and 75 mol.% This is 29 and 38% higher than in the control and even more significantly higher by 134 and 93%, compared with the number of cells on similar samples of films, but processed in a continuous mode. Similarly, the highest number of fibroblasts is recorded on all films of P(3HB-co-3HHx) copolymers, regardless of the content of medium-chain 3HHx monomers. The number of cells in this variant is 49–50% higher than the control and 100% higher than the result on films of these types processed in the continuous mode of laser radiation.

In contrast to the results of the MMT test obtained in the study of films treated in the quasi-pulse mode, the number of viable fibroblasts grown on films after continuous laser irradiation decreases significantly, including in comparison with the number of cells on the original untreated films and, in general, is close to the control (number of cells on polystyrene) (Figure 7). The lowest number of cells is on films obtained from P(3HB) and P(3HB-co-3HV) copolymers. Somewhat higher is the number of cells on films obtained from two other types of copolymers, P(3HB-co-4HB) and P(3HB-co-3HHx). On average and in general terms, the reduction in the number of fibroblasts on films processed in a continuous mode, compared with untreated films, is at least 20%, maximum 37 and 45%.

Thus, the number of metabolically active fibroblasts and those grown on the studied polymer films is most pronouncedly affected by the laser treatment mode and, to a lesser extent, by the composition of PHAs.

In general, we can state a more favorable effect on the development of fibroblasts of the studied line of films of all the studied types of PHAs after treatment of films with a CO_2_ laser in a quasi-pulse mode. This confirms the results obtained earlier on the example of three types of copolymer PHAs films (P(3HB-co-3HV), P(3HB-co-4HB), and P(3HB-co-3HHx) with one content of second monomers at the level of 30 mol.%) [50].

As is known, laser processing is considered one of the most suitable methods for modifying the surface of cell matrices. This is due to the fact that laser processing makes it possible to form surface reliefs of complex shape, while providing high structuring accuracy, allowing for the creation of complex biomedical devices with high biocompatibility without the use of toxic chemicals [84,85].

The positive effect of laser treatment on the development of eukaryotic cells has been described in a series of papers. Thus, the modification of films from the P(3HB-co-3HV) copolymer using Nd:YAG laser treatment provides stronger attachment and development of keratinocytes on the matrix compared to the original films [48]. A similar positive effect has been obtained on an example of flexible films made of P(3HB-co-3HV) irradiated by ultraviolet laser treatment in a culture of human keratinocytes [49]. The use of a krypton fluoride (KrF) excimer laser for processing P(3HB-co-3HV) films has been positively evaluated in cultures of mouse fibroblasts (NIH 3T3) and bone osteosarcoma cells U-2 OS [58]; the authors have noted the formation of depressions on the surface and an increase in roughness. Films of P(3HB) as well as other polymers (PLA, PMMA, and PU/PDMS) treated with an excimer laser enhance adhesion and proliferation of human fibroblasts in culture [60].

Similar positive results of laser processing have been described for other polymers. For example, femtosecond laser treatment of thin films of chitosan and a composition of chitosan with ceramics favorably influences the attachment and development of osteoblasts isolated from the calvaria of mice and mesenchymal stem cells of adipose tissue [86]. The favorable effect of the use of a femtosecond laser for surface treatment of poly(L-lactide)/hydroxyapatite films on the development of human osteoblast ATCC is described in [87]. A study by [88] shows that femtosecond treatment of biodegradable PLLA films with the formation of perforations on the surface improves adhesion, growth, and development of myoblasts, and promotes their proliferation and differentiation. E. Rebollar with colleagues [89] has been presented periodic surface structures generated by linearly polarized KrF laser light (248 nm) on polystyrene (PS) foils; these structures have a periodicity of 200 to 430 nm and a depth of 30 to100 nm. The surface laser modification results in a significantly enhanced adhesion and proliferation of human embryonic kidney cells (HEK-293) compared to the unmodified polymer foil. Furthermore, the authors have reported on the alignment of HEK-293 cells, Chinese hamster ovary (CHO-K1) cells, and skeletal myoblasts along the direction of the structures. The results indicate that the presence of nanostructures on the substrates can guide cell alignment along definite directions, and more importantly, in our opinion, that this alignment is only observed when the periodicity is above a critical periodicity value that is cell-type specific.

Thus, multicenter studies include (1) a set of films obtained from four types of PHAs (10 variants); (2) including copolymer films (nine variants) are obtained from three different types of PHAs with different ratios of monomers; (3) all versions of the films are treated with two different laser radiation regimes.

**Figure 8:**
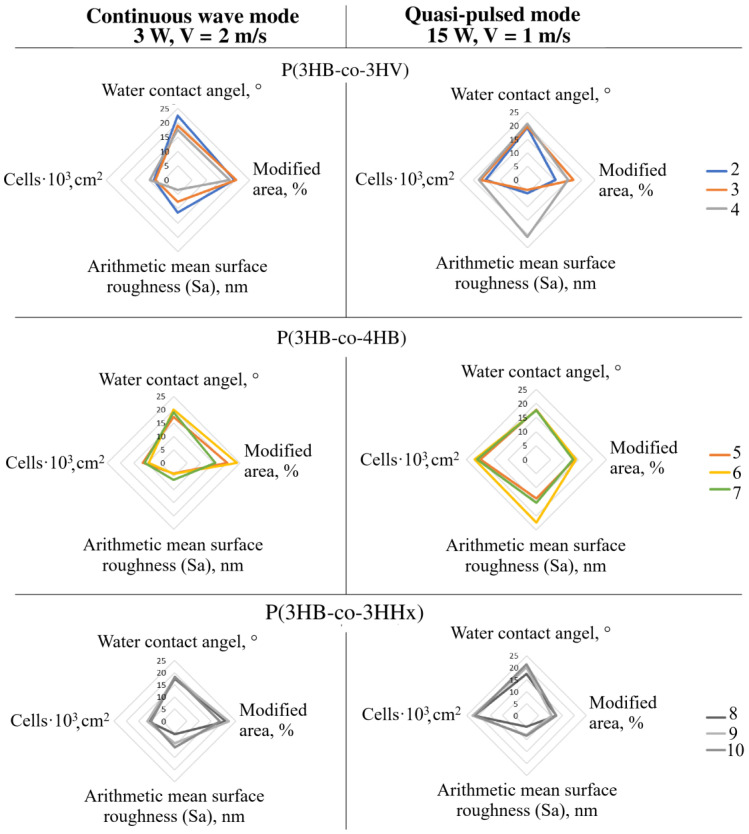
Radar chart representation of the characteristic values of PHAs films processed in 2 laser irradiation modes, (numbering—Table 1): 2–4—P(3HB-co-3HV); 5–7—P(3HB-co-4HB); 8–10—P(3HB-co-3HHx).

Due to the multifactorial nature of the objects of study and indicators for assessing the consequences of laser radiation, an integrated approach has been used to statistically process the results obtained in the form of plotted radar diagrams to characterize the complex effect of two types of laser processing on the total modified surface area of the films, the average roughness, the value of the contact angle, and the number of viable cells, according to the MTT test (Figure 8). This type of radar diagram is used in such studies [90,91].

In these works, the use of such a mathematical model makes it possible to reveal the regularities of the complex multifunctional impact of the studied processing mechanisms on the properties of the resulting products.

It can be seen that for all film samples processed in the quasi-pulse regime of laser radiation, which is accompanied by a reduction in the total modified surface and an increase in the arithmetic mean roughness for a group of samples from P(3HB-co-4HB) and a sample of P(3HB-co-3HV) with a maximum inclusion of 3HV and a decrease in this parameter in samples from P(3HB-co-3HV) with low and medium inclusions of 3HV monomers, it increases the biological compatibility of polymer films and provides an increase in the number of physiologically active fibroblasts. In contrast to these results, the use of continuous laser radiation is accompanied by an increase in melted modified areas. At the same time, it is not observed that there are changes in the average roughness and the value of the contact angle between the samples. This is accompanied by a decrease in the biological compatibility of the surface of irradiated polymer films and the number of viable fibroblasts. From this point of view, the use of this analysis of the results in the form of radar diagrams makes it possible to more correctly identify indicators that positively affect the biological compatibility of cellular matrices.

It can be seen that for the films treated in the quasi-pulse regime of laser radiation, which is accompanied for all samples by reduced values of the total modified surface and an increase in the arithmetic mean roughness for a group of samples from P(3HB-co-4HB) and a sample of P(3HB-co-3HV) with a maximum incorporation of 3HV, and a decrease in roughness in the P(3HB-co-3HHx) group and samples from P(3HB-co-3HV) with low and medium incorporation of 3HV monomers, biocompatibility increases and an increase in the number of physiologically active fibroblasts is provided. In contrast to these results, the use of continuous laser radiation is accompanied by an increase in melted modified areas and, in general, the area of modifications. In this case, no significant changes in the contact angle between the samples are observed, with the exception of the P(3HB-co-3HV) copolymer with low 3HV inclusion and the P(3HB-co-4HB) copolymers with medium and high 4HB inclusions, and while processing these samples quasi-pulse mode reduces the values of the contact angle. This is accompanied by a decrease in the biological compatibility of the surface of the irradiated polymer films and the number of viable fibroblasts. The use of this analysis of the results in the form of radar diagrams makes it possible to more correctly identify indicators that positively affect the biological compatibility of cell matrices.

## 4. Conclusions

For the first time, the properties of solvent casting films obtained from PHAs, not only with a different set of monomers, but with a different ratio of them, the surface of which is modified by CO_2_ laser irradiation in continuous or quasi-pulsed modes, have been studied. The PHAs samples used to prepare the films are P(3HB) homopolymer and three types of copolymers formed by 3-hydroxybutyrate monomers with 4-hydroxybutyrate, or 3-hydroxyvalerate, or 3-hydroxyhexanoate. The ratio of monomers «3HB:other types» varies from 75:15 to 25:75 mol.%

The consequences of laser treatment of the surface of films obtained from various types of PHAs includes the study of the structure of the surface microrelief (SEM and AFM), roughness indices, water contact angles, adhesion, and fibroblast growth. The consequences of laser treatment are determined depending on the radiation regime and the composition of monomers in PHAs. The surface modification after continuous treatment consists in the formation of melted areas in the form of grooves, the total area of which is 11% of the total area of films for P(3HB) and from 12 to 15% for copolymer films. The quasi-pulse mode by the raster method causes the formation of holes without pronounced melted zones, the total area of which is lower by 20% compared to the area of melted grooves. The processing mode affects the surface properties, cell adhesion, and fibroblast growth. The number of viable fibroblasts on the films after the quasi-pulse mode is 1.5–2.0 times higher compared to the continuous mode, and depends to a greater extent on the laser treatment mode than on the PHAs’ composition.

The presence of PHAs of various compositions and the use of continuous and quasi-pulsed laser radiation regimes make it possible to influence the morphology and surface properties of polymer films in a targeted manner. Changes in the structure and properties of the surface of the studied polymer films obtained from PHAs of various compositions, with different ratios of monomers and irradiated with two different laser treatment modes, in our opinion, should be considered today as preliminary results requiring special theoretical studies, as well as additional experimental studies. The results make it possible for direct modification of the surface of polymer products made from PHAs, a promising family of biodegradable polymers.

## Figures and Tables

**Table 1:** Characterization of PHAs different composition.

Sample	PHAs Composition (mol.%)	Average Molecular Weight Mw (kDa)		Polydispersity Ð	Degree of Crystallinity Cx (%)	Glass Transition Temperature Tg (°C)	Crystallization Temperature Tc (°C)	Melting Temperature Tmelt (°C)	Thermal Degradation Temperature Tdegr (°C)
P(3HB)—3-hydroxybutyrate homopolymer									
1	100.0	920		2.5	78	-	85	176.3	280.2
Copolymers:									
P(3HB-co-3HV)									
2	85.0	15.0	690	2.8	65	−1.0	64.2	144.4	272.1
3	72.8	27.2	576	3.2	54	−1.9	78.1	162.5	275.9
4	35.0	65.0	398	3.44	58	−4.4	57.0	148.6	278.6
P(3HB-co-4HB)									
5	86.0	14.0	715	2.9	44	−11.7	68.2	161.4	272.3
6	64.5	35.5	660	3.6	22	−9.5	58.5	165.5	278.4
7	24.8	75.2	840	3.8	16	−3.6	78.1	169.2	260.1
P(3HB-co-3HHx)									
8	91.0	9.0	520	3.9	60	−0.2	63.2	170.2	262.7
9	83.6	16.4	390	4.3	49	−0.6	57.2	168.7	281.5
10	62.0	38.0	486	3.7	52	−1.6	71.2	169.2	260.1

«-»—not determined.

**Table 2:** Characterization of the surface structural elements of laser-treated films prepared from PHAs with different compositions.

Continuous Wave Mode, 3 W, V = 2 m/s					
Sample	PHAs Composition, mol.%		Width of Grooves, µm	Distance between Grooves, µm	Modified Area, %
P(3HB)—3-hydroxybutyrate homopolymer					
1	100.0		115.8 ± 5.6	890.2 ± 5.3	11.5 ± 0.8
Copolymers:					
P(3HB-co-3HV)					
2	85.0	15.0	132.7 ± 4.4	878.3 ± 5.4	13.1 ± 0.6
3	72.8	27.2	140.2 ± 2.9	891.4 ± 5.9	13.6 ± 0.5
4	35.0	65.0	122.6 ± 3.7	896.6 ± 2.3	12.0 ± 0.5
P(3HB-co-4HB)					
5	86.0	14.0	135.6 ± 3.3	875.6 ± 5.1	13.4 ± 0.5
6	64.5	35.5	163.3 ± 6.5	864.2 ± 7.8	15.9 ± 1.0
7	24.8	75.2	105.5 ± 13.0	906.4 ± 15.5	10.4 ± 1.8
P(3HB-co-3HHx)					
8	91.0	9.0	145.5 ± 2.1	884.7 ± 2.0	14.1 ± 0.3
9	83.6	16.4	152.5 ± 6.0	872.3 ± 5.3	14.9 ± 0.8
10	62.0	38.0	125.1 ± 3.8	889.6 ± 1.9	12.3 ± 0.5
**Quasi-pulsed mode, 15 W, V = 1 m/s**					
**Sample**	**PHAs Composition,** **mol.%**		**Pit Diameter, µm**	**Distance between** **Pits, µm**	**Modified area, %**
P(3HB)—3-hydroxybutyrate homopolymer					
1	100.0		151.3 ± 8.5	346.8 ± 19.2	7.2 ± 1.0
Copolymers:					
P(3HB-co-3HV)					
2	85.0	15.0	145.9 ± 4.1	343.9 ± 21.0	7.0 ± 0.8
3	72.8	27.2	192.0 ± 19.0	313.7 ± 17.5	11.3 ± 1.4
4	35.0	65.0	177.0 ± 14.9	320.4 ± 19.2	9.9 ± 1.3
P(3HB-co-4HB)					
5	86.0	14.0	172.0 ± 18.2	335.9 ± 17.7	9.00 ± 1.3
6	64.5	35.5	174.8 ± 17.9	332.2 ± 18.8	9.3 ± 1.3
7	24.8	75.2	167.6 ± 26.4	336.0 ± 26.9	8.7 ± 1.5
P(3HB-co-3HHx)					
8	91.0	9.0	161.1 ± 15.1	335.4 ± 18.1	8.3 ± 1.2
9	83.6	16.4	149.0 ± 17.3	348.5 ± 18.0	7.0 ± 1.2
10	62.0	38.0	160.5 ± 27.3	333.5 ± 20.9	8.3 ± 1.5

**Table 3:** Surface roughness parameters of the laser-treated films prepared from PHAs samples, based on results of atomic force microscopy (AFM).

Sample	PHAs Composition, Monomer Content (mol.%)		Continuous Wave Mode3 W, V = 2 m/s			Quasi-Pulsed Mode 15 W, V = 1 m/s		
			Arithmetic Mean Surface Roughness (Sa) nm	Root Mean Square Roughness (Sq) nm	Peak-to-Valley Height (Sz) nm	Arithmetic Mean Surface Roughness (Sa) nm	Root Mean Square Roughness (Sq) nm	Peak-to-Valley Height (Sz) nm
P(3HB)—3-hydroxybutyrate homopolymer								
1	100.0		232.5	282.2	1682.0	198.7	245.8	1226.3
Copolymers								
P(3HB-co-3HV)								
2	85.0	15.0	228.0	294.5	1802.4	99.3	122.8	689.9
3	72.8	27.2	152.1	202.7	1437.4	73.5	94.1	598.2
4	35.0	65.0	69.6	84.2	496.4	419.5	486.2	2284.9
P(3HB-co-4HB)								
5	86.0	14.0	78.1	104.2	1084.3	275.8	442.3	5135.2
6	64.5	35.5	83.4	106.3	671.6	446.1	561.5	2542.5
7	24.8	75.2	126.2	148.5	761.5	305.5	388.4	2612.1
P(3HB-co-3HHx)								
8	91.0	9.0	108.9	133.7	657.9	92.8	117.5	735.1
9	83.6	16.4	182.1	221.7	1457.5	159.8	188.8	869.9
10	62.0	38.0	216.5	251.9	1079.7	169.9	202.1	972.9

## Data Availability

Not applicable.
